# Photosensitization of TiO_2_ nanofibers by Ag_2_S with the synergistic effect of excess surface Ti^3+^ states for enhanced photocatalytic activity under simulated sunlight

**DOI:** 10.1038/s41598-017-00366-7

**Published:** 2017-03-21

**Authors:** Samina Ghafoor, Sadia Ata, Nasir Mahmood, Salman Noshear Arshad

**Affiliations:** 1 Department of Chemistry, Syed Babar Ali School of Science and Engineering, Lahore University of Management Sciences, Lahore, 54792 Pakistan; 2 Institute of Chemistry, University of the Punjab, P.O. Box 54590, Lahore, Pakistan; 3 Institute for Superconducting and Electronic Materials, Australian Institute for Innovative Materials, University of Wollongong, North Wollongong, 2500 Australia

## Abstract

TiO_2_ nanofibers, with mean diameter ~200 nm, were fabricated by electrospinning and successfully photosensitized with low bandgap Ag_2_S nanoparticles of 11, 17, 23 and 40 nm mean sizes, with corresponding loading of 4, 10, 18 and 29 wt.% Ag_2_S, respectively. 17 nm Ag_2_S@TiO_2_ nanofibers exhibited optimal activity in the photodegradation of methylene blue under simulated sunlight with pseudo-first order rate constant of 0.019 min^−1^ compared to 0.009 min^−1^ for pure TiO_2_ nanofibers. In spite of greater visible-light absorption and reduced bandgap, larger than 17 nm Ag_2_S nanoparticles exhibited sluggish photodegradation kinetics probably due to less photo-induced carriers generation in TiO_2_ and reduced electron injection rates from the larger sized Ag_2_S into TiO_2_. Furthermore, a UV-O_3_ surface treatment induced excess Ti^3+^ surface states and oxygen vacancies which synergistically enhanced the photodegradation rate constant to 0.030 min^−1^ for 17 nm Ag_2_S@TiO_2_ sample which is ~70% better than the previously reported for Ag_2_S/TiO_2_ hierarchical spheres. This was attributed to the efficient charge separation and transfer driven by increased visible-light absorption, bandgap narrowing and reduced electron-hole recombination rates. The present study demonstrate the potential utilization of Ag_2_S@TiO_2_ nanofibers in filtration membranes for removal of organic pollutants from wastewater.

## Introduction

Hazardous wastewater pollution is becoming a serious threat to human health^1^ which necessitates the development of green and efficient water remediation technologies. Visible-light driven photocatalysis using suitable bandgap oxide-based nanomaterials is an effective technique to chemically transform the organic pollutants into non-hazardous compounds^2^. Titanium dioxide (TiO_2_) is widely used as a photocatalyst because it is non-toxic, chemically and thermally stable and inexpensive with a strong oxidizing ability^3–5^. However, its practical use is limited by very low visible-light activity and high recombination rates of photo-induced electron-hole pairs^6^. The Anatase phase of TiO_2_ has a bandgap (E_g_) of 3.2 eV which covers only 3–5% of the solar spectrum. Moreover, TiO_2_ is commonly used as nanoparticles (NPs) which exhibits low photodegradation rates for many organic pollutants due to agglomeration and low active area loading. Electrospinning is a relatively easy and low cost technique for synthesis of ultra-long TiO_2_ nanofibers (NFs) and permeable membranes^7^ which can facilitate the charge transportation and separation, thus, reducing the recombination rate of electron-hole pairs^8, 9^. In fact, Choi *et al*. has shown that TiO_2_ NFs exhibit superior photocurrent generation (factor of ~3) and hydrogen production (factor of ~7) compared to TiO_2_ NPs due to well-ordered and interconnected architecture and inherent meso-porosity which aids in adsorption and desorption of the reactants and products, respectively^10^. Similarly, enhanced photocatalytic activity of electrospun mesoporous TiO_2_ nanofibers was demonstrated by CO_2_ reduction to renewable hydrocarbon fuels^11^.

Many attempts have been made to enhance the photocatalytic activity of TiO_2_ under illumination conditions similar to solar light^12, 13^. These include doping transition metal ions such as Fe^3+^ ^14, 15^, Ni^2+^ & Ti^3+^ ^16^, Ti^3+^-doped TiO_2_^17, 18^ or nonmetal atoms such as N, F and S^19–21^, dye-sensitization^22–24^, and coupling with a photosensitizer such as g-C_3_N_4_^25^, MFe_2_O_4_^26^, α-Fe_2_O_3_, Au, Pt and Au/Pt co-catalysts^27–29^, CdS^30, 31^, MoS_2_@zeolite^32^, Ag^33^ and Ag_3_PO_4_^34, 35^. The purpose of coupling with a photosensitizer is to enhance the visible-light harvesting and promote charge separation by effective interface charge transfer between the photosensitizer and TiO_2_ for which the relative position of the energy band levels are critical. Recently, Ag_2_S has gained considerable attention as a photosensitizer for TiO_2_^36–41^. Ag_2_S is a direct low bandgap semiconductor material (E_g_~1.0 eV) which responds well in the whole solar energy spectrum. The conduction (CB) and valence (VB) bands of Ag_2_S are −0.3 eV and +0.7 eV, respectively, which is very desirable for photosensitization of TiO_2_ with corresponding CB and VB of −0.1 eV and +3.1 eV, respectively^36^. Zhang *et al*. demonstrated hollow Ag-Ag_2_S/TiO_2_ composite spheres with superior photocatalytic activity in reduction of Cr (VI) under both UV and visible-light^42^. Gholami *et al*. reported 15 fold enhancement of photocurrent for free standing TiO_2_ nanotube array, each of 125 nm diameter and 4.1 μm length, sensitized by ~17 nm Ag_2_S NPs^37^. Shan *et al*. demonstrated core-shell Ag/Ag_2_S NPs on TiO_2_ nanowires as a model photoelectrochemical electrode system^38^. Ong *et al*. used sequential ionic deposition to fabricate Ag_2_S NPs on TiO_2_ hierarchical spheres. Enhanced simulated solar light driven photocatalytic performance was demonstrated by water splitting with hydrogen production at 708 μmolh^−1^ g^−1^ and photodegradation of methyl orange with pseudo-first order rate constant of 0.018 min^−1^ ^39^.

Immobilization of Ag_2_S NPs on TiO_2_ nanofibers (NF) is expected to give higher number of active sites. Multiple factors contribute to the photosensitization effect of Ag_2_S on TiO_2_ nanofibers such as (a) Effect of Ag_2_S mean size and loading amount on the injection rate of photo-induced electrons into TiO_2_ as well as photo-induced carriers generation rate in TiO_2_, (b) Effect of Ag_2_S mean size, loading and coverage on the light harvesting capability, (c) Effect of chemical interaction between Ag_2_S and TiO_2_ on possible lowering of the energy bandgap and the recombination rate, and (d) Effect of changing the surface chemical states and defects on TiO_2_ (e.g. Ti^3+^ and oxygen vacancies) on the carrier separation and transport. Li *et al*.^43^ deposited different amounts of Ag_2_S NPs on TiO_2_ nanorod arrays by varying the number of cycles in successive ionic layer absorption and reaction (SILAR) method. They reported an optimum 25 cycle deposition for maximum photocatalytic degradation rates of methylene orange (MO) under visible-light. However, other factors as noted above were not investigated in detail.

In this report, the combined effect of all these factors for photosensitization of TiO_2_ nanofibers has been experimentally investigated and optimized. We demonstrate optimal simulated solar light driven photocatalytic activity for TiO_2_ NFs sensitized by ~17 nm Ag_2_S NPs, loaded at 10 wt.%, with the synergistic effect of enhanced Ti^3+^ chemical states and oxygen vacancies induced by a facile UV-O_3_ surface treatment. Moreover, these composite nanofibers were stable over multiple cycles of photodegradation and demonstrate their potential use for water remediation.

## Results and Discussion

### Morphology, structure and chemical composition

**Figure 1:**
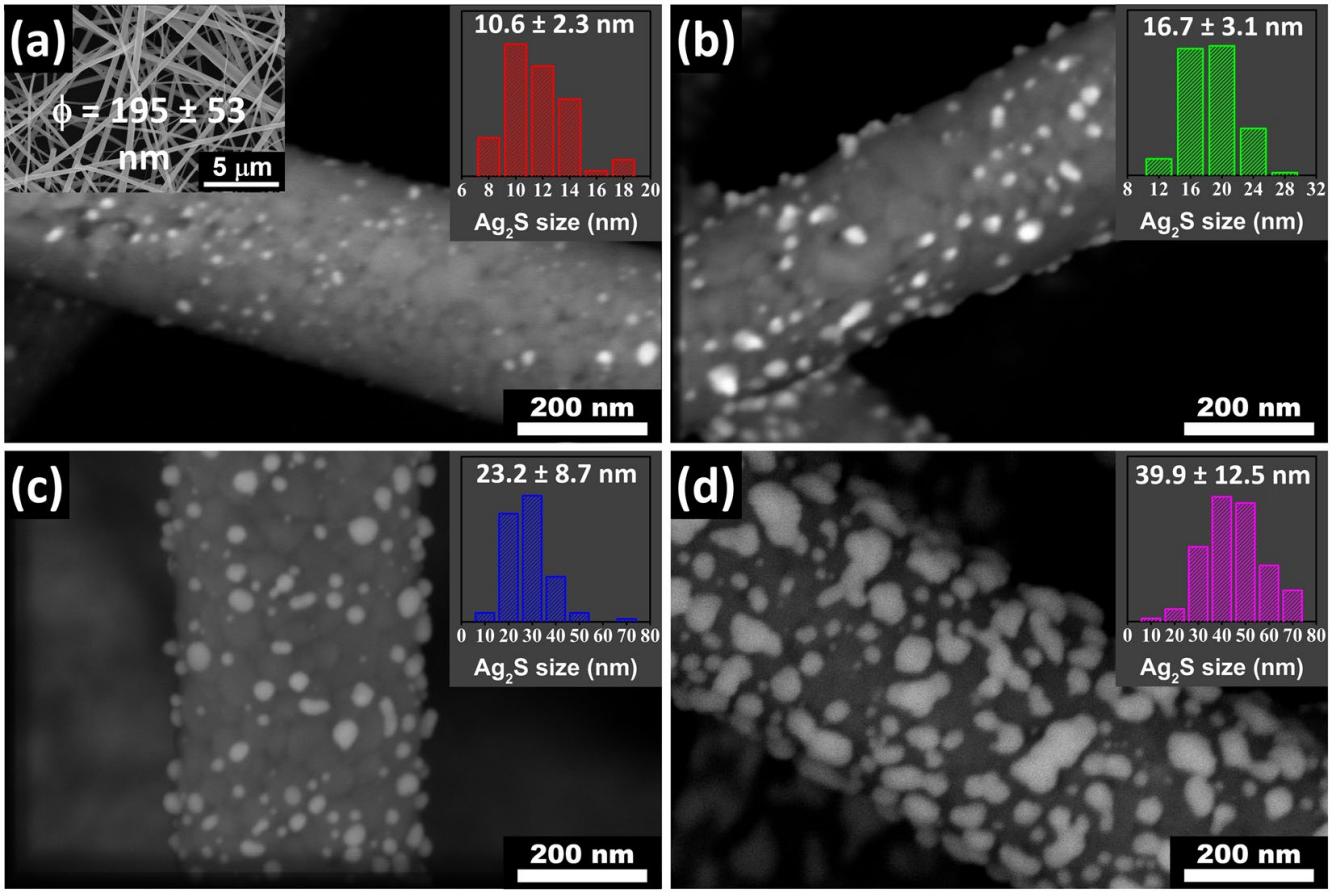
High resolution SEM images of Ag_2_S@TiO_2_ NFs with mean Ag_2_S size of (**a**) 11 nm, (**b**) 17 nm, (**c**) 23 nm and (**d**) 40 nm. The Ag_2_S size distribution is shown in the insets. A low magnification SEM image of the TiO_2_ NFs is also shown as inset in (**a**).

The morphology with mean sizes and distribution of Ag_2_S@TiO_2_ NFs are shown in Fig. 1. The uncoated TiO_2_ NFs (Fig. 1(a) inset) have a mean diameter of 195 nm and interstices of ~1 μm size, formed by the interpenetrating nanofibers network. The color of the TiO_2_ NFs changed instantaneously as they were dipped in the Tollens reagent with addition of 0.05 M dextrose solution indicating the formation of Ag NPs followed by washing with distilled water and ethanol. The mean size of the Ag NPs was controlled by varying the dipping time in the solution from 0.5–4 min. Figure 1(a–d) shows the successful deposition of Ag_2_S NPs by nucleation and growth of Ag followed by sulfurization without any structural damage to TiO_2_ NFs. The phase and chemical composition of the Ag_2_S NPs was confirmed by XPS and XRD as discussed later. The sizes of Ag_2_S NPs showed characteristically log-normal distribution with mean number-averaged size of 11, 17, 23 and 40 nm for dipping times of 0.5, 1, 2 and 4 min, respectively. The number-averaged sizes were statistically measured by tracing at least 100 particles, computing their areas, A, and calculating the corresponding area-equivalent diameters, D, assuming spherical shape $(D=\sqrt{4A/\pi })$. The particles remain distinct and approximately spherical for dipping times up to 2 min but most of them coalesce into bigger and mostly irregular particles after 4 min dipping. In this study, the samples will be labelled as x nm-Ag_2_S@TiO_2_ where ‘x’ is the mean size of the Ag_2_S NPs. The weight percent loading of Ag_2_S in the composite nanofibers was determined by inductively coupled plasma optical emission spectroscopy. A standard AgNO_3_ salt solution of 1–10 ppm was used to calibrate the emission intensity of Ag which fitted linearly with R^2^ value of 0.999. The weight fraction of 11, 17, 23 and 40 nm Ag_2_S@TiO_2_ composite nanofibers was found to be 4, 10, 18 and 29%, respectively, assuming that all the Ag converted to Ag_2_S.

**Figure 2:**
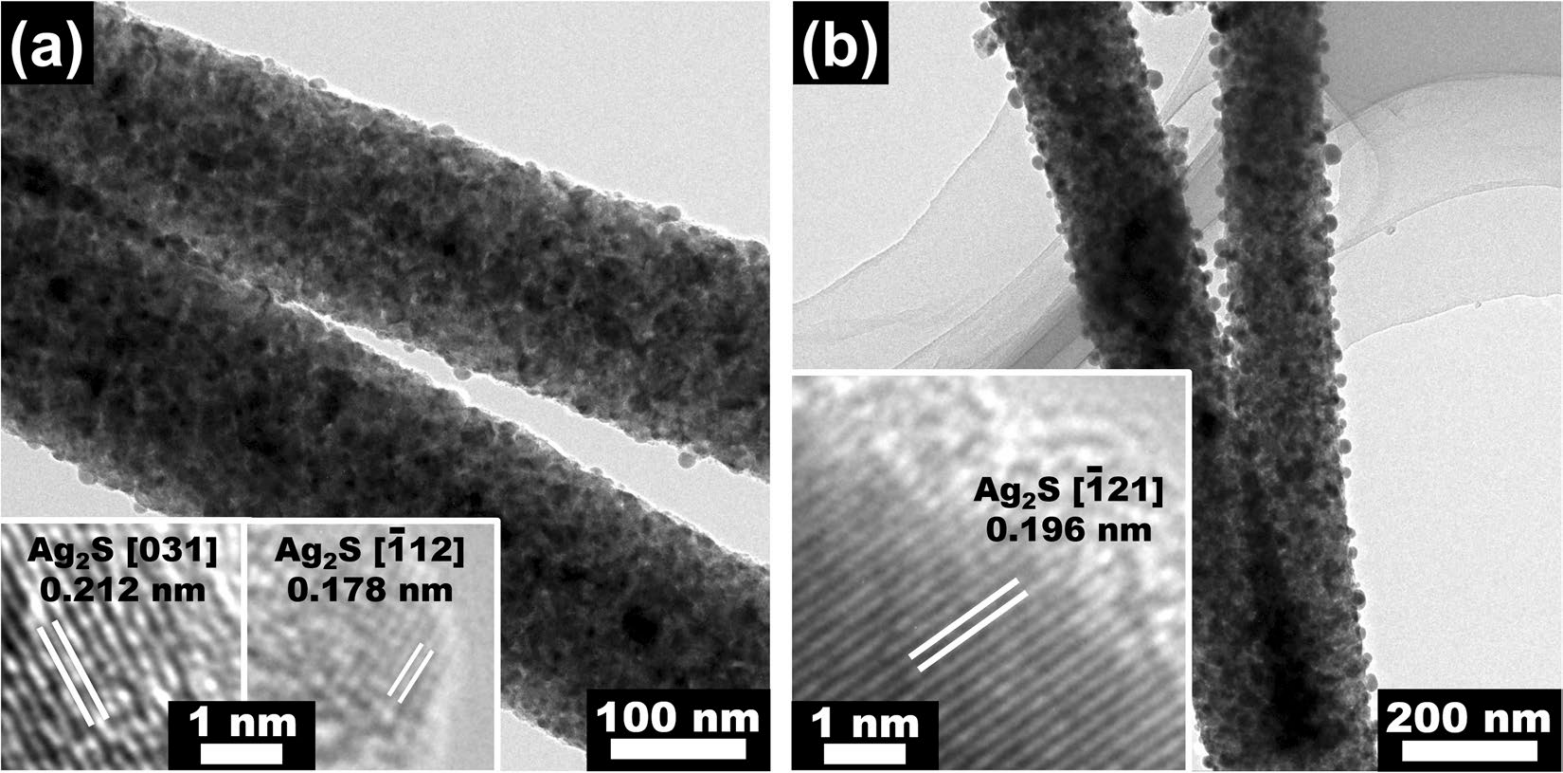
TEM images of (**a**) 11 nm-Ag_2_S@TiO_2_ and (**b**) 23 nm-Ag_2_S@TiO_2_ NFs. Insets are HR-TEM images near the nanofiber edges showing the lattice fringes of the Ag_2_S NPs.

**Figure 3:**
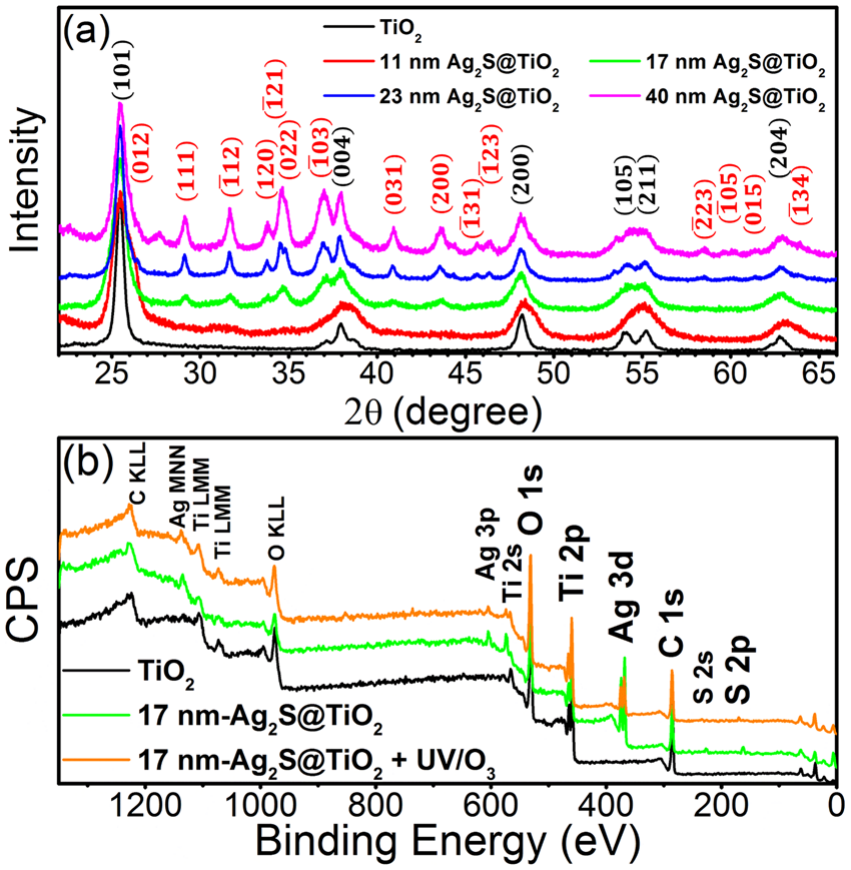
(**a**) XRD scans of pure TiO_2_ and Ag_2_S@TiO_2_ NFs. (**b**) Full XPS survey scan of pure TiO_2_, 17 nm-Ag_2_S@TiO_2_ and 17 nm-Ag_2_S@TiO_2_ + UV-O_3_ samples.

TEM images of 11 nm- and 23 nm-Ag_2_S@TiO_2_ NFs in Fig. 2(a and b) also show uniform deposition of Ag_2_S NPs on the NF surfaces. Their sizes are comparable to those measured from the SEM images. The insets show HR-TEM images of Ag_2_S NPs near the NF edges. The lattices identified in 11 nm-Ag_2_S@TiO_2_ correspond to (031) and $(\bar{1}12)$ crystallographic planes with d-spacing of 0.212 and 0.178, respectively, whereas the lattice in 23 nm-Ag_2_S@TiO_2 _correspond to $(\bar{1}21)$ crystallographic plane with d-spacing of 0.196 nm. These were also confirmed by the powder XRD scans of Ag_2_S@TiO_2_ NF samples shown in the Fig. 3(a), where $(\bar{1}12)$, $(\bar{1}21)$ and (031)^44^ signals were easily detected (JCPDS # 14-0072, Ag_2_S, monoclinic). The most intense peak at 2θ = 25.3° corresponds to the (101) crystallographic plane and confirms the formation of pure Anatase TiO_2_ phase in all the samples^45^. The chemical composition was verified by the full scan XPS of TiO_2_, 17 nm-Ag_2_S@TiO_2_ and 17 nm-Ag_2_S@TiO_2_ + UV-O_3_ samples as shown in the Fig. 3(b) which confirms the presence of Ti and O in the un-coated samples and additionally, Ag and S, in the coated samples. All the samples showed C 1 s signal which is typical of the adsorbed surface contaminations. The detailed XPS analysis, discussed next, confirmed the presence of Ag_2_S on the NF surfaces.

**Figure 4:**
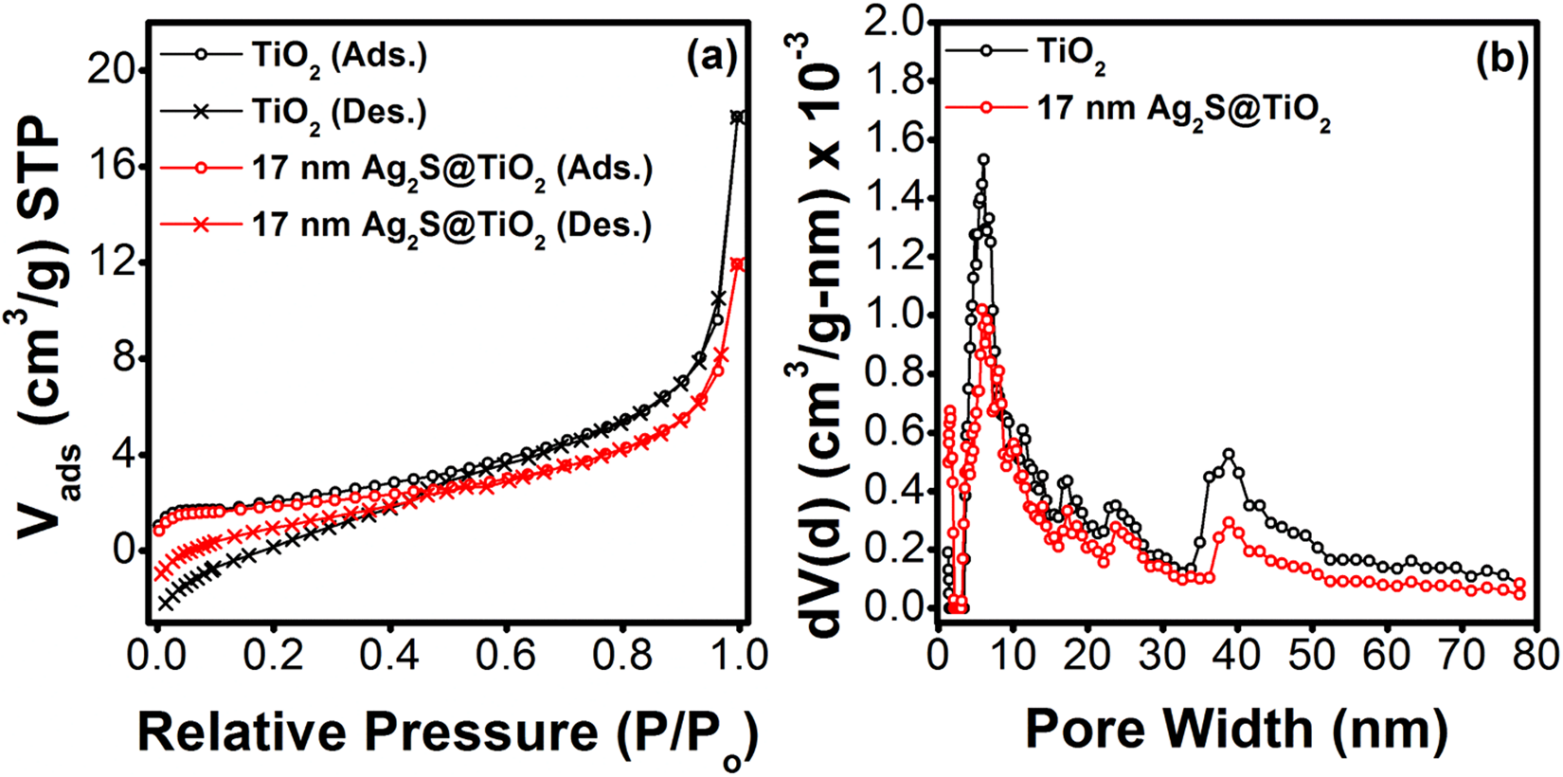
(**a**) Nitrogen adsorption/desorption isotherms for pure TiO_2_ NFs and 17 nm-Ag_2_S@TiO_2_ composite nanofibers, and (**b**) the corresponding pore size distributions.

Adsorption-desorption isotherm of pure TiO_2_ NFs and 17 nm-Ag_2_S@TiO_2_ composite NFs in Fig. 4(a) closely resembles a type-II isotherm which is typical for non-porous materials due to multilayer formation. The specific surface area of pure TiO_2_ NFs and 17 nm-Ag_2_S@TiO_2_ composite NFs was 7.1 and 6.6 m^2^/g, respectively, which is consistent with the reported value of 13.3 m^2^/g for control TiO_2_ nanofibers^46^. The pore size distribution in Fig. 4(b) was calculated by NLDFT method. Absence of hysteresis on desorption branch rules out the presence of mesopores, however, high intake of nitrogen at P/P_0_ > 0.8 could be attributed to the presence of macropores. Contribution of micropores to the total surface area as calculated by the t-plot method was found to be almost zero.

### Surface chemical states of Ag_2_S@TiO_2_ nanofibers

**Figure 5:**
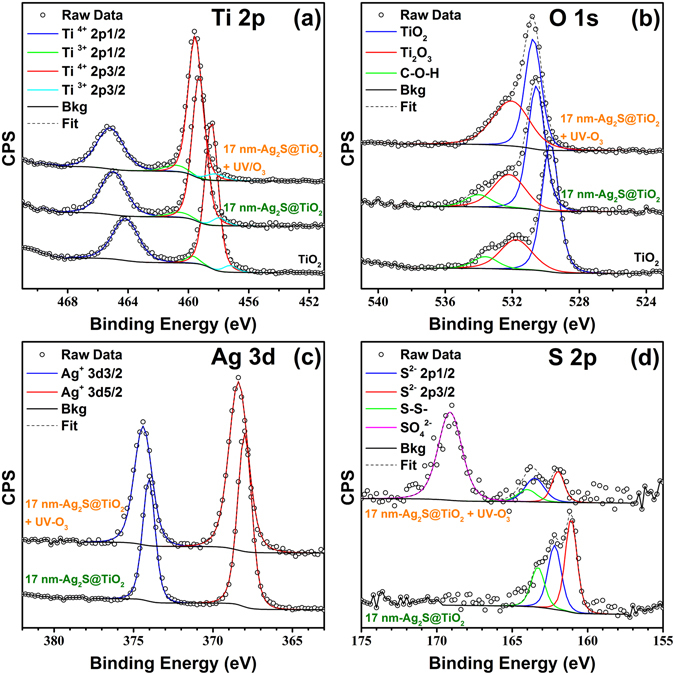
XPS spectra of pure TiO_2_, 17 nm-Ag_2_S@TiO_2_ and 17 nm-Ag_2_S@TiO_2_ + UV-O_3_ samples. (**a**) Ti 2p, (**b**) O 1 s, (**c**) Ag 3d, and (**d**) S 2p.

The effect of photosensitization by Ag_2_S NPs and UV-O_3_ treatment on the surface chemical states were determined by XPS. Ti 2p signal of pure TiO_2_ NFs had two peaks at 464.1 and 458.4 eV which corresponds to Ti 2p_1/2_ and Ti 2p_3/2_ spin-orbit splitting, respectively, as shown in the Fig. 5(a). According to the Handbook of X-ray Photoelectron Spectroscopy^47^, the binding energies of Ti 2p_1/2_ and Ti2p_3/2_ in TiO_2_ are 464.3 and 458.8 eV, respectively. The area ratio of these two peaks is ~0.5 (33.67/66.32 = 0.507) with a binding energy difference of ~5.7 eV (464.15–458.46 = 5.69 eV) which is in good agreement with the literature^48^. Ti 2p signal was deconvoluted into four peaks which showed 94.3% Ti^4+^ and 5.7% Ti^3+^ oxidation state on the TiO_2_ NFs surface. The Ti^3+^ oxidation state increased to 6.7% after coating with 17 nm Ag_2_S NPs which confirms the lower electron density of Ti atoms in Ag_2_S@TiO_2_ NFs. Additionally, the Ti 2p peak positions shifted to higher binding energy indicating the withdrawal of valence electron charge (i.e. oxidation) owing to the strong interaction between Ag_2_S and TiO_2_ NFs^49^. The UV-O_3_ treatment further shifted the Ti 2p peaks towards higher binding energies with increase in Ti^3+^ surface state to 8.2% which indicates the removal of the lattice oxygen. This increase in surface Ti^3+^ had a significant effect on the photocatalytic activity as discussed later.

Deconvolution of O 1 s spectra of TiO_2_ NFs gave three peaks at 529.7, 531.8 and 533.6 eV, as shown in the Fig. 5(b), which are associated with O^2−^ species in the lattice (TiO_2_), oxygen vacancies or defects (Ti^3+^ or Ti_2_O_3_) and chemisorbed or adsorbed oxygen species, respectively^50^. Similar to Ti 2p, the peaks shifted to higher binding energies after coating with Ag_2_S NPs and UV-O_3_ treatment. The Ti_2_O_3_ content increased from 27.4 to 30.2% after coating with 17 nm Ag_2_S NPs and further to 47.7% after the UV-O_3_ treatment. These results suggest that oxygen vacancies and excess electrons in Ti are generated by this process which results in the reaction Ti^4+^ → Ti^3+^ near the surface.

In the Ag 3d XPS spectra, shown in Fig. 5(c), two peaks were observed at 368.0 eV and 374.0 eV representing 3d_5/2_ and 3d_3/2_ spin-orbit pair, respectively. These peaks were shifted to 368.4 eV and 374.4 eV after UV-O_3_ surface treatment and indicated the presence of Ag in Ag^+^ form^39, 51^. The S 2p XPS spectra in Fig. 5(d) showed peaks at 161.09 eV and 162.19 eV representing 2p_3/2_ and 2p_1/2_ of S^2−^ in the metal sulfides, thus, confirming the successful formation of Ag_2_S. Another smaller peak at 163.3 eV is assigned to the -S-S- bonding^52^ probably due to chemisorption and presence of excess elemental sulfur. These results are in good agreement with the oxidation state of Ag_2_S in the literature^53^. Again, these peaks are slightly shifted after UV-O_3_ surface treatment with emergence of another peak at 169.12 eV due to SO_4_^2−^ formation by the surface oxidation of sulfur.

### Photocatalytic performance and mechanism

**Figure 6:**
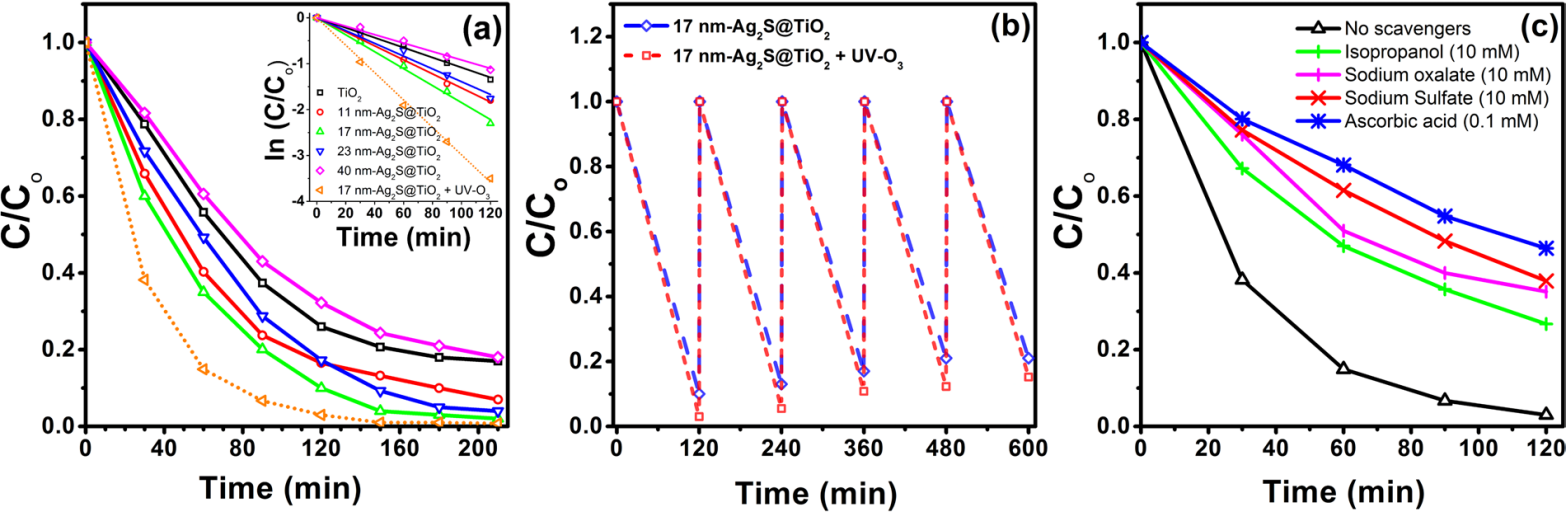
(**a**) Comparative photocatalytic performance (C/C_o_) of pure and Ag_2_S sensitized TiO_2_ NFs under simulated solar light irradiation (inset: pseudo-first order kinetics), (**b**) Photocatalytic performance of 17 nm-Ag_2_S@TiO_2_ NFs over 5 cycles, and (**c**) Photocatalytic performance in the presence of radical scavengers.

The Ag_2_S mean size, coverage of TiO_2_, and UV-O_3_ surface treatment had a profound effect on the photocatalytic performance. The photocatalytic activity of Ag_2_S@TiO_2_ NFs were evaluated by monitoring the photodegradation of MB. In a typical experiment, 5 mg of the composite NFs was dispersed in 10 ml of 10 ppm MB solution and kept in dark for 30 min under continuous stirring to attain a complete adsorption-desorption equilibrium^54^. The suspension was then illuminated by a 100 W Xenon arc lamp and photodegradation monitored by the UV-Vis spectrophotometer by measuring the absorbance of MB solution at regular time intervals. The C/C_o_ as a function of the irradiated time, where C_o_ is the initial MB solution concentration after the adsorption-desorption equilibrium has been attained, is shown in the Fig. 6(a). All samples showed pseudo-first order photodegradation kinetics with a rate constant of 0.011 min^−1^ for pure TiO_2_ NFs which increases to 0.015 min^−1^ and 0.019 min^−1^ after photosensitization by 11 nm and 17 nm Ag_2_S NPs, respectively. Remarkably, the rate constant significantly increases to 0.030 min^−1^ after a 2 hr UV-O_3_ surface treatment on 17 nm-Ag_2_S@TiO_2_ sample which is ~70% better than 0.018 min^−1^ reported for Ag_2_S NPs on TiO_2_ hierarchical spheres^39^. This was attributed to the generation of excess Ti^3+^ surface states and oxygen vacancies, as shown by the XPS results, which facilitated the charge separation process and reduced the electron-hole recombination. We previously reported enhanced photocatalytic activity under UV irradiation for TiO_2_ NPs (P25 Degussa) loaded polyacrylonitrile NFs due to excess Ti^3+^ surface defects and enhanced hydrophilicity induced by a UV-O_3_ surface treatment^55^. These NFs showed stable performance when tested over 5 cycles of photodegradation as shown in the Fig. 6(b). Please note that the slight decrease in performance is attributed to ~10% material weight loss during the repeated separation and re-use over 5 cycles. The stability of the samples after photodegradation was investigated by SEM and XRD. The phase, size and loading remained stable with no signs of leaching as shown in the Supplementary Information Fig. 1s.

The radical trapping experiments were carried out to identify the main active oxidation species for photodegradation of MB. As shown in the Fig. 6(c), the photocatalytic efficiencies of the composite nanofibers significantly decreased from 97% to 54%, 62%, 65%, and 73% after the addition of ascorbic acid (as O_2_^−•^ scavenger), sodium sulfate (as e^−^ scavenger), sodium oxalate (as h^+^ scavenger), and isopropanol (as ^•^OH scavenger), respectively. This indicates that the photoinduced electrons react with dissolved O_2_ molecules to yield O_2_^−•^ radicals, while the positively charged holes (h^+^) react with OH^−^ derived from H_2_O to form ^•^OH radicals. Thus, all these play a significant role in the photodegradation of MB.

### Optical properties of Ag_2_S@TiO_2_ nanofibers

**Figure 7:**
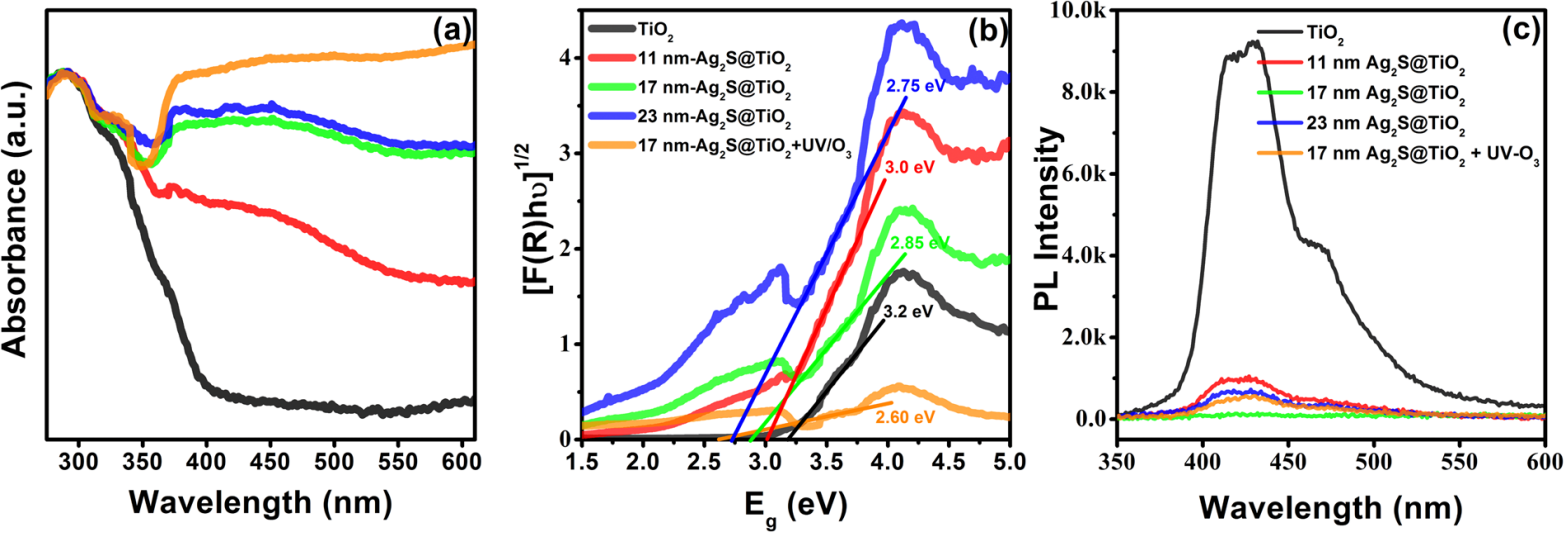
(**a**) UV-vis diffuse reflectance spectra of Ag_2_S@TiO_2_ NFs (converted to absorbance), (**b**) Kulbelka-Munk transformation showing the estimated bandgaps, and (**c**) the photoluminescence spectra recorded after 325 nm laser excitation.

**Figure 8:**
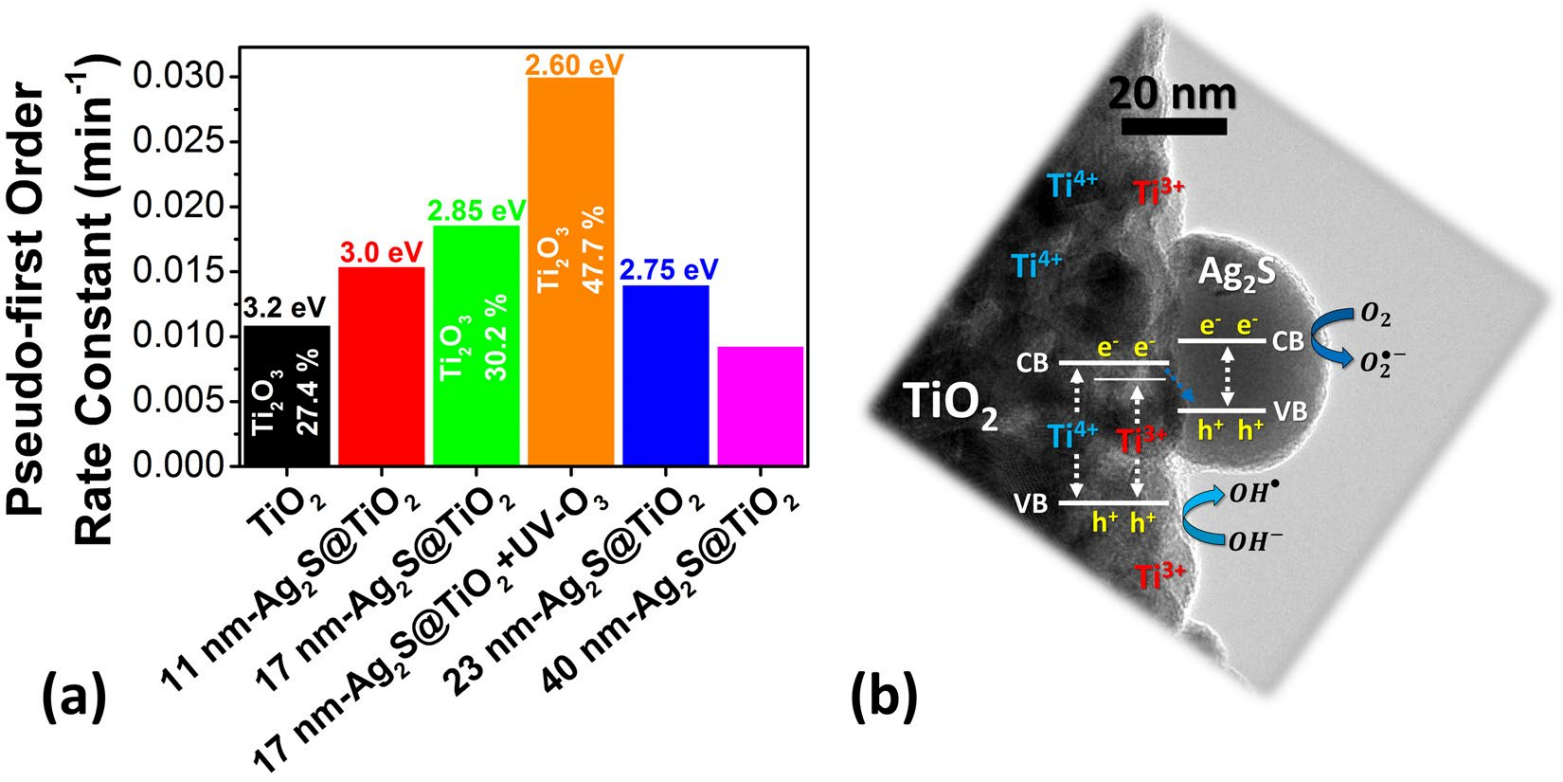
(**a**) Methylene blue photodegradation rate constants for all the samples. Percentage of Ti_2_O_3_ surface states (estimated form O 1 s XPS signal) and calculated bandgaps are also listed. (**b**) Schematic of the interfacial charge transfer processes during illumination of Ag_2_S sensitized TiO_2_ nanofibers with excess Ti^3+^ states and oxygen vacancies.

To investigate the optical response, the absorption spectra of the Ag_2_S@TiO_2_ NFs and their corresponding Kulbelka-Munk transformation of the reflectance spectra are shown in Fig. 7(a and b), respectively. Pure TiO_2_ showed the expected bandgap of 3.2 eV and very weak visible-light absorption. However, the visible-light absorption increased and the bandgap decreased, monotonically, by coating with Ag_2_S NPs of increasing size due to the enhanced interfacial chemical interaction and coverage of TiO_2_ by the smaller bandgap (E_g_~1 eV) Ag_2_S generating new midgap energy levels. However, the increased size of Ag_2_S will have a negative impact on the photo-induced carriers generation rate in TiO_2_ and injection rate of electrons from the conduction band of Ag_2_S into TiO_2_, thus, increasing their recombination probability. This was shown by the 23 nm- and 40 nm-Ag_2_S@TiO_2_ samples which, despite having a lower bandgap (E_g_~2.75 eV), showed even less photocatalytic activity then pure TiO_2_ NFs with rate constants of 0.014 and 0.009 min^−1^, respectively, as shown in the Fig. 8(a). To investigate the promoted charge separation, the samples were excited by 325 nm laser which generates electron-hole pairs. The recombination of these photoexcited carriers gives photoluminescence (PL) signal with an intensity directly proportional to the electron-hole recombination process. Thus, a lower PL is desirable as it indicates a lower charge recombination which will result in a higher photocatalytic activity. As shown in the Fig. 7(c), the pure TiO_2_ NF showed a high PL intensity around 425 nm which significantly diminished for Ag_2_S photosensitized TiO_2_ NFs. Similar PL results are reported for Ag/TiO_2_^56^ and Ag_2_O/TiO_2_^57^ systems.

### The synergistic effect of photosensitization and surface Ti^3+^ states

The enhanced photocatalytic performance of 17 nm-Ag_2_S@TiO_2_ + UV-O_3_ sample is attributed to the synergistic effect of photosensitization by Ag_2_S NPs and excess surface Ti^3+^ states and oxygen vacancies. The proposed effective charge transfer process between Ag_2_S and TiO_2_ due to the preferred conduction band (CB) and valence band (VB) positions of Ag_2_S, which are lower than corresponding bands in TiO_2_, is schematically depicted in the Fig. 8(b). This facilitated the generated electrons transfer from CB of Ag_2_S to the CB of TiO_2_ to effectively inhibit the electron-hole recombination. This photosensitization effect is further supplemented by the high Ti_2_O_3_ (Ti^3+^ state and oxygen vacancies) near the surface which leads to enhanced absorption of incident solar light below the bandgap of TiO_2_ (Ti^4+^ state) as shown in the Fig. 8(b) and reported in ref. 58. The midgap state is close to the CB which facilitates the injection of photogenerated electrons into TiO_2_ under simulated solar light. Moreover, the Ti^3+^ defects and oxygen vacancies slows down the electron-hole recombination kinetics by acting as hole traps, thus, prolonging the lifetime of the charges^59^.

In summary, photosensitization of electrospun TiO_2_ nanofibers was done by Ag_2_S nanoparticles with mean sizes of 11–40 nm range for enhanced simulated solar light driven photocatalytic performance. Detailed morphological and structural characterization by SEM, HR-TEM & XRD and chemical states identification by XPS confirmed the growth of Ag_2_S nanoparticles. The key characteristics and parameters of all the samples are shown in the Table 1. The size and coverage of Ag_2_S had significant effect on the photocatalytic activity with 17 nm-Ag_2_S@TiO_2_ giving the optimal activity. This was further enhanced by a surface UV-O_3_ treatment which introduced excess Ti^3+^ surface defects and oxygen vacancies and reduced the bandgap. This synergistic effect of Ag_2_S sensitization and UV-O_3_ treatment is attributed to the reduced recombination effect due to efficient charge transfer. The prepared composite NFs were stable in performance when tested over 5 cycles with a maximum photodegradation rate constant of 0.03 min^−1^ for the UV-O_3_ treated 17 nm-Ag_2_S@TiO_2_ nanofibers which is ~70% better than the previously reported value for Ag_2_S@TiO_2_ hierarchical spheres. These results demonstrate the potential of using these composite nanofibers for water remediation under sunlight.

**Table 1:** Material characteristics and photodegradation rate constants for all the samples.

Samples	Ag_2_S mean size (nm)	Ag_2_S wt.%	Ti^3+^ (Ti 2p)	Ti^3+^ (O 1 s)	Bandgap (eV)	Rate Constant (min^−1^)
TiO_2_	—	—	5.7	27.4	3.2	0.011
Ag_2_S@TiO_2_ (t_dip_ = 0.5 min)	10.6 ± 2.3	4	—	—	3.0	0.015
Ag_2_S@TiO_2_ (t_dip_ = 1.0 min)	16.7 ± 3.1	10	6.7	30.2	2.85	0.019
Ag_2_S@TiO_2_ (t_dip_ = 1.0 min) +UV-O_3_ treated			8.2	47.7	2.60	0.030
Ag_2_S@TiO_2_ (t_dip_ = 2.0 min)	23.2 ± 8.7	18	—	—	2.75	0.014
Ag_2_S@TiO_2_ (t_dip_ = 4.0 min)	39.9 ± 12.5	29	—	—	—	0.009

## Methods

### Fabrication of TiO_2_ nanofibers

To fabricate TiO_2_ NFs by electrospinning, 1.5 g polyvinylpyrrolidone (PVP, M_w_ = 1,300,000 g/mol) was mixed in 10 g ethanol and 4 g acetic acid under vigorous stirring for 1 hr. Then 4 g Titanium(IV) n-butoxide (TNBT, 97%) was added in the solution and further stirred for 1 hr to generate a homogeneous electrospinning solution. This precursor solution was electrospun at 18 kV electrical potential with tip to collector distance of 18 cm with pumping rate of 0.8 mL/hr. The non-woven NF mat was left on the aluminum wired collector overnight for complete hydrolysis and annealed in air at 500 °C for 2 hr with a heating rate of 5 °C/min to obtain pure Anatase TiO_2_ NFs.

### Fabrication of Ag_2_S@TiO_2_ nanofibers

Ag_2_S NPs of different size distribution were grown on TiO_2_ NFs by a two-step process: reduction of Ag^+^ to Ag followed by sulfurization of Ag to Ag_2_S. Typically, 0.1 M NaOH was added drop-wise in 10 mL of 0.01 M AgNO_3_ solution until appearance of brown precipitates. Then 1.0 M ammonia solution is added drop-wise until dissolution of all brown precipitates which indicates the formation of silver-ammonia complex [Ag(NH_3_)_2_]^+^, commonly known as the Tollens reagent. 10 mL of 0.05 M dextrose solution and 20 mg TiO_2_ NFs were added into this freshly prepared solution under magnetic stirring, resulting in the formation of Ag NPs. These Ag NPs were then sulfurized to Ag_2_S^40^ by dipping in 25 mL of 0.01 M sulfur/acetonitrile solution at 60 °C for 10 min. The Ag_2_S@TiO_2_ NFs were then washed with acetonitrile and distilled water and then dried at 60 °C in oven. The selected Ag_2_S@TiO_2_ NFs were also exposed to UV-O_3_ for 2 hr in a UV-Ozone plasma cleaner chamber.

### Characterization

The size and morphology of Ag_2_S NPs and TiO_2_ NFs was characterized by field emission scanning electron microscopy (FE-SEM, FEI 450 Nova NanoSEM) and high resolution transmission electron microscopy (HR-TEM, Jeol JEM-2100F operating at 200 kV). The weight fraction of Ag_2_S in the composite nanofibers was determined by inductively coupled plasma – optical emission spectroscopy (ICP-OES, Perkin Elmer Optima 2100 DV). 3 mg of the sample was digested in 5 mL conc. HNO_3_ and then diluted up to 50 ml with deionized water. The surface chemical composition and oxidation states of Ti, O, Ag and S were quantified by X-ray photoelectron spectroscopy (XPS, PHI-1600) equipped with Al Kα radiation. The peak positions were calibrated to C 1 s (284.8 eV) and plotted using the CasaXPS software. The photoluminescence spectra of the composite nanofibers were recorded by exciting the samples by 325 nm wavelength laser. The crystalline phases were determined by powder X-ray diffraction (XRD, Bruker D2 Phaser). Diffuse reflectance spectra (DRS) in 200–800 nm range were taken by UV-vis-NIR spectrophotometer (Jasco, V-770) to study the light harvesting capabilities of the samples and estimate their bandgaps using Kubelka-Munk function. The specific surface area was measured using Quantachrome (NOVA 2200 e) surface area and pore size analyzer. Silica kernel with cylindrical pore morphology and NLDFT (equilibrium mode) were used for the calculation of pore volume and pore size. Surface area was measured from nitrogen adsorption-desorption isotherm by applying B.E.T. plot in relative pressure range of 0.05–0.30 P/P_o_.

### Photocatalytic performance

The photocatalytic performance of the Ag_2_S@TiO_2_ NFs were evaluated by photodegradation of methylene blue (MB) solution in water. In a typical experiment, 5 mg of the sample was dispersed in a 10 mL solution of 10 ppm MB and kept in dark for 30 min under continuous stirring to attain a complete adsorption-desorption equilibrium on the surface of the composite NFs. Photocatalytic degradation was carried out by illuminating the suspension with simulated solar light using a commercial 100 W Xenon lamp. The analytical sample from the photocatalytic reaction was collected at regular time intervals and centrifuged. The resulting solution was analyzed by recording the absorbance of the residual MB solution with UV-vis spectrophotometer at a wavelength of 662 nm. To identify the active oxidation species responsible for the photocatalytic degradation of MB, a series of radical trapping experiments were carried out. Isopropanol as ^•^OH scavenger (10 mM), ascorbic acid as O_2_^−•^ scavenger (0.1 mM), sodium sulfate as e^−^ scavenger (10 mM) and sodium oxalate as h^+^ scavenger (10 mM) were added directly into the MB solution containing the composite photocatalysts prior to irradiation by simulated solar light.

## Electronic supplementary material

Supplementary Information
