# Supplementary material for: Photosensitization of TiO2 nanofibers by Ag2S with the synergistic effect of excess surface Ti3+ states for enhanced photocatalytic activity under simulated sunlight
Source: Sci Rep. 2017 Mar 21;7:255. doi: 10.1038/s41598-017-00366-7 (PMC5428275; doi:10.1038/s41598-017-00366-7)
Supplement: Supplementary file 1 — Supplementary Information [file 41598_2017_366_MOESM1_ESM.doc]

**Supplementary Information**

**Photosensitization of TiO_2_ nanofibers by Ag_2_S with synergistic effect of excess surface Ti^3+^ states for enhanced photocatalytic activity under simulated sunlight**

Samina Ghafoor,^a,b^ Sadia Ata,^b^ Nasir Mahmood,^c^ Salman Noshear Arshad ^*,a^

^a^ Department of Chemistry, Syed Babar Ali School of Science and Engineering, Lahore University of Management Sciences, Lahore 54792, Pakistan.

^b^ Institute of Chemistry, University of the Punjab, P.O. Box 54590, Lahore, Pakistan.

^c^ Australian Institute for Innovative Materials, University of Wollongong, Northfields Ave, Wollongong NSW 2522, Australia.

^*^ Corresponding author: salman.arshad@lums.edu.pk

**
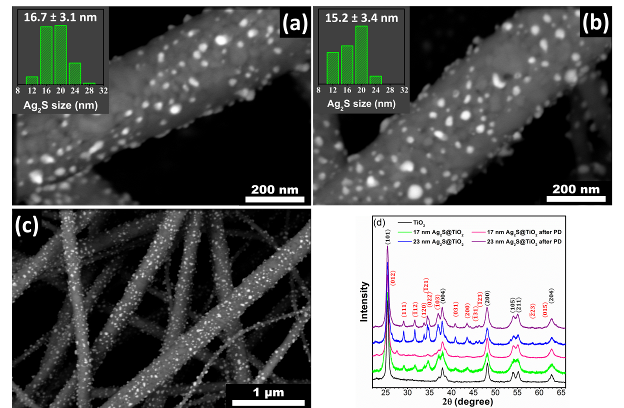
**

**Fig. 1s** High resolution SEM images of the as-prepared 17 nm Ag_2_S@TiO_2_ NFs (a) before and (b) after photo-degradation experiments, (c) A low magnification SEM image of (b) showing even coating and distribution of Ag_2_S nanoparticles without any signs of leaching. Moreover, the mean size of Ag_2_S remains consistent before and after photodegradation within one-half of the standard deviation. (d) XRD scans of as-prepared pure TiO_2_, 17 and 23 nm Ag_2_S@TiO_2_ NFs before and after photodegradation. A slight decrease in the intensity after photodegradation for Ag_2_S is attributed to the texture effects because these samples are repeatedly handled and pressed during photocatalytic experiments and the subsequent removal and sample preparation for XRD. Otherwise, SEM images shows no sign of leaching and degradation of the composite photocatalysts.
